# Nitrogen sources affected the biosynthesis of 2-acetyl-1-pyrroline, cooked rice elongation and amylose content in rice

**DOI:** 10.1371/journal.pone.0254182

**Published:** 2021-07-15

**Authors:** Pouwedeou Mouloumdema Potcho, Nnaemeka Emmanuel Okpala, Tchalla Korohou, Muhammad Imran, Nabieu Kamara, Jisheng Zhang, Kelvin Dodzi Aloryi, Xiangru Tang

**Affiliations:** 1 State Key Laboratory for Conservation and Utilization of Subtropical Agro-Bioresources, College of Agriculture, South China Agricultural University, Guangzhou, China; 2 Scientific Observing and Experimental Station of Crop Cultivation in South China, Ministry of Agriculture and Rural Affairs, Guangzhou, China; 3 College of Engineering, Nanjing Agricultural University/Key Laboratory of Intelligent Agricultural Equipment of Jiangsu Province, Nanjing, China; 4 State Key Laboratory for Conservation and Utilization of Subtropical Agro-Bioresources, South China Agricultural University, Guangzhou, China; 5 Sierra Leone Agricultural Research Institute (SLARI), Freetown, Sierra Leone; Bangabandhu Sheikh Mujibur Rahman Agricultural University, BANGLADESH

## Abstract

Many studies have been carried out on N sources effect on fragrant rice; however, their impact on rice grain quality is largely unclear. In this study, we evaluated the effects of different types of N sources on rice growth, yield, 2-acetyl-1-pyrroline (2AP), amylose and cooked rice elongation. Two indica rice cultivars, Basmati 385 (B385), Xiangyaxiangzhan (XYXZ) and two japonica cultivars, Yunjingyou (YJY), Daohuaxiang (DHX) were grown in experimental pots with six replications under four N sources: Potassium nitrate (KNO_3_), ammonium bicarbonate (NH_4_HCO_3_), urea (H_2_NCONH_2_) and sodium nitrate (NaNO_3_) in 2019 and 2020 early seasons. Our results showed that N dynamics regulated the number of panicles, 1000-grain weight, grain yield, 2-acetyl-1-pyrroline, amylose and cooked rice elongation across all the four treatments. The NH_4_HCO_3_ treatment significantly increased the number of panicles and grain yield across the four rice varieties compared with KNO_3_, H_2_NCONH_2_ and NaNO_3_ N sources in both 2019 and 2020 early season, The KNO_3_ treatment significantly showed higher 1000-grain weight in B-385, YJY, XYXZ and DHX compared to other N sources. Compared with other N sources treatment, the NH_4_HCO_3_ treatments significantly increased the 2AP contents in heading stage leaves, matured leaves and grains of B-385, YJY, XYXZ and DHX respectively. Cooked rice elongation percentage also showed significant difference in all treatments studied with KNO_3_ recorded the highest across the four varieties. Analysis of major enzymes and compounds such as P5C, P5CS, PDH, Pyrroline, proline and Methylglyoxal showed remarkable differences in each cultivar at heading and maturity stages with higher activity in NH_4_HCO_3_ and H_2_NCONH_2_ treatments. Similarly, in all treatments, we also observed significant increase in amylose content percentage, with NH_4_HCO_3_ having greater percentage of amylose.

## Introduction

The main concern today on a global scale, is to minimize the use of nitrogen in the crops production under climate change condition and the efficient use of nitrogen is recognized as an important production factor for rice [1]. A large amount of chemical fertilizers, especially nitrogen (N) has played an important role in the advancement of rice production in the past [2]. However, excess N fertilizer accompanied by low efficiency of nutrient utilization is a serious problem in rice production all over the world, as it affects yield and grain quality [3,4].

Previous studies on the effect of fertilizer application on yields, milling and quality parameters of aromatic rice have mainly focused on the role of an individual nutrient [5]. Park et al. (2019) [6] suggested that, the aroma, sweetness, whiteness, stickiness and glassiness of cooked milled rice are inversely proportional to the percentage of nitrogen in the cereals and it has been argued that the application of 10 t/ha of farmyard manure or 80 or 120 kg of N ha^-1^ increased the head of rice recovery and alkaline value of fragrant rice varieties. In addition, nitrogen fertilizer application increased grain length, length: width ratio, grain length after cooking, and aspect ratio. The researcher also suggested that potassium fertilizer should be applied at the rate giving the highest grain yield to produce the best combination of yield and aromatic rice quality. The influences of nitrogen fertilizers on the quality of the grain are due to the effects on the activity of the biosynthetic carbohydrate enzymes. Also, by the same way, these nitrogen fertilizers would influence the synthesis of amylose [7]. For various growth and development processes, plants absorb nitrogen (N) in the form of nitrate (NO_3_^−^) or ammonium (NH_4_^+^). The NO_3_ form is mobile, less toxic and can be stored in vacuoles for most crop plants; however, NO_3_^-^ must be reduced to NH_4_^+^ before it can be used [8].

2-aceytl-1-pyrroline (2AP) with the chemical formula C6H9NO and popcorn-like flavor has been reported to be the main compound responsible for the flavor of rice and it has been reported in all parts of cultivars of fragrant rice, except in the roots [9–14]. For its unique ’popcorn’ or ’nutty’ flavor, fragrant rice is a special subgroup of rice widely distributed around the world [12,15]. The strong expression of fragrance plays an important role in the marketing of rice and Consumer demand for fragrant rice is increasing around the world [16,17]. The 2AP accumulation is affected by nitrogen application and Yang et al. (2012) [18] reported that, high total nitrogen content led to a high flavor content of cereals. [19] found that 2AP content in grains was increased with increasing nitrogen application. Field crops generally take up potassium (K) faster than nitrogen (N) or phosphorus (P), where K also plays an important role in ensuring efficient nitrogen use with an input of KNO_3_ for example [20]. Thus the K input plays a role in many important regulatory processes in the plant such as the quality of the rice grain related to 2AP [20]. Ammonium bicarbonate (NH_4_HCO_3_), Nitrate (NO_3_), Urea (H_2_NCONH_2_) and other compound fertilizers are important and can affect crop growth, yields and therefore quality through the known effect of nitrogen on 2AP [21,22]. For example, Yang et al. (2012) [18] reported that ammonia bicarbonate could regulate the activities of protective enzymes in rice. They have also proved the osmotic effect of Na^+^ on the roots via the supply of NaNO_3_.

The distribution of amino acids, proteins and the length of the amylopectin chain are largely affected by nitrogen fertilizer in rice as an example [23]. Previous studies have suggested that proline is an important precursor for 2AP formation [14,24]. In addition, Δ 1-pyrroline-5-carboxylate (P5C), Δ 1pyrroline-5-carboxylate synthetase (P5CS), and proline dehydrogenase (PDH) are highly related to the biosynthesis of 2AP [25,26]. The content of 2AP in cereals has become one of the characteristics used to assess the quality of fragrant rice more recently. More and more scientists are studying different methods to increase its concentration in grains [19]. In higher plants, ornithine, proline, and glutamate can be converted to a common metabolite, Δ 1 -pyrroline-5-carboxylic acid, via three distinct enzymes: ornithine aminotransferase (OAT), proline dehydrogenase (PRODH), and Δ 1 -pyrolline-5-carboxylic acid synthetase (P5CS) [14]. The same author has shown that Δ 1 -pyrolline-5-carboxylic acid could react with Methylglyoxal to form 2-acetyl-1-pyrroline.

Rice fragrance [27], cooked rice elongation [28] and amylose content [29] are some of the traits used in determining rice grain quality. However, very few studies have been conducted on the relationship between these sources of N: KNO_3_, NH_4_HCO_3_, H_2_NCONH_2_ and NaNO_3_, their effect on the pathway of 2AP biosynthesis and the quality of rice in general. In addition, studies on the effect of these sources of nitrogen (KNO_3_, NH_4_HCO_3_, H_2_NCONH_2_ and NaNO_3_) on the enzymes involved in the biosynthesis of 2AP, amylose content and cooked rice elongation are still very rare.

The aim of this study, therefore, is to evaluate the effect of N sources on the biosynthesis of 2AP, cooked rice elongation percentage and amylose content percentage. We also carried out comparative studies on the impact of N sources, during rice growth and development.

## 2. Materials and methods

### 2.1. Plant materials and experimental details

Two indica rice cultivars: Basmati 385 (B385) and Xiangyaxiangzhan (XYXZ) and two japonica rice cultivars: Yunjingyou (YJY) and Daohuaxiang (DHX) were used for the study. Rice cultivars were obtained from the College of Agriculture, South China Agricultural University (SCAU) Guangzhou-China. Before sowing under greenhouse conditions, the seeds were soaked in water for 24 h at room temperature (25°C). Then, the pre-germinated seeds were hill-seeded. These experiments were conducted from March, 2019 to July, 2019 and from March, 2020 to July, 2020 at the Experimental Research Farm, College of Agriculture, South China Agricultural University, China (23°09’ N, 113°22’ E and 11 m from mean sea level). The sprouted seeds were sown in PVC trays for the nursery, then placed in a puddled field and covered with a sheet of plastic. Seedlings were transplanted into a plastic pot of 31 cm in diameter and 29 cm in height containing 10 kg sandy loam soil. The soil-filled pots with four hills per pot and three seedlings per hill were transplanted with uniform and stable rice seedlings of 22-days old.

### 2.2. Experimental designs and treatments

Four compounds: Potassium nitrate (KNO_3_ with molar masse (M) = 101.10 and CAS No = 7757-79-1), Urea (H_2_NCONH_2_ with M = 60.06 and CAS No = 57-13-6), Sodium nitrate (NaNO_3_ with M = 84.99 and CAS No = 7631-99-4), and Ammonium bicarbonate (NH_4_HCO_3_ with M = 79.06 and CAS No = 1066-33-7) were used as N sources for this experiment. Damao Chemical Reagent Factory has provided all these nitrogen sources.

N percentages of each compound are as follows: KNO_3_ (13.854%) H_2_NCONH_2_ (46.65%) NaNO_3_ (16.48%) and NH_4_HCO_3_ (17.718%).The treatments were arranged under completely randomized design (CRD) with six replicates. We determined the mass of nitrogen per 100g of each chemical compound used in this experiment by using the following formula.

$$\text{MN}=\frac{\text{N}\times 100\;\text{g}}{\text{mmol}}$$

Where MN is the mass of nitrogen per 100 g, N denotes the atomic molar mass of nitrogen and mmol is the molar mass of each compound. Thus, in 100 g of each fertilizer namely, KNO_3,_ H_2_NCONH_2,_ NaNO3 and NH_4_HCO_3_, nitrogen dose was 13.85 g, 23.30 g, 16.48 g and 17.70 g respectively. To have the needed dose we added the equivalent of 2 g of nitrogen of each compound in a pot i.e. KNO_3_ = 14.45 g, H_2_NCONH_2_ = 8.59 g, NaNO_3_ = 12.14 g and NH_4_HCO_3_ = 11.29 g. Each pot received 1 g of KH_2_PO_4_ (with M = 136.09 and CAS No = 7778-77-0 provided by **Damao Chemical Reagent Factory**) to make available the Potassium (K) and phosphorus (P) which are very essential for the growth of rice plants. However, the first half 50% of all the four N sources and 1 g of KH_2_PO_4_ were applied in each pot at the basal before transplanting and the second half 50% at the tillering stage.

### 2.3. Sampling and data collection

We collected data such as: the number of tillers by counting the tillers from their appearance for each treatment and each variety until the total appearance of the panicles, then an average was made per pot after reducing the total number of seedlings (12) transplanted and dividing with the number of hills in each pot. The number of panicles was also counted by monitoring their appearance until maturity and an average per pot was made. Leaves (green) were collected during the heading stage and harvest time. When harvesting, grains were collected from each treatment. The fresh leaves and the grains were separated then immediately immersed in liquid nitrogen and stored in ice box to be transported to the laboratory and stored immediately at -80°C until biochemical analyses. After sun drying, grain samples for amylose content percentage cooked rice elongation percentage were left at room temperature (25°C) for three months before dehusking and milling and 2AP content grain samples were left at -80°C before analysis.

### 2.4. GC-MS analysis for the determination of 2-acetyl-1-pyrroline

GC-MS analysis was performed with GCMS-QP 2010 (GCMS-QP 2010 Plus, Shimadzu Corporation, Japan), with the following specifications: The SH-Rxi-5Sil MS chromatographic column was 30 m long, 0.25 mm in diameter, 0.25 μL of film thickness and the heating temperature at its peak was 220°C; Helium—high purity of 99.999% was used as carrier gas; a constant pressure division less injection method was used; the injection volume was 2 μL; the mass spectrometry used was the electron bombardment ion source, the ion source temperature was 200°C; the ionization energy was 70 eV; the interface temperature was 250°C; the quadrupole temperature was 150°C; full scan mode with m/z 35–160 scan mass range. The samples used in this study had three replications. Each replication was prepared with 2 g of powdered sample. The ground sample was transferred to a 20 mL bottle, followed by the addition of 10 mL of dichloromethane (CH_2_Cl_2_). The samples were then transferred to an ultrasonic cleaner (KQ-800ES from KUNSHAN ULTRASONIC INSTRUMENT CO.LTD.), set at 0°C for 4 h. Then the samples were transferred to a 10 mL conical flask before the addition of 4 g of anhydrous sodium sulfite (Na_2_SO_3_). After lowering for 30 seconds, 1 mL of the supernatant was transferred to a vial with a disposable micropipette. This was followed by the addition of 2 μL of 2,3,6-trimethylpyridine (1000 x dilution with CH_2_Cl_2_) as an international standard. The vials were closed and transferred to the GC-MS machine for analysis. The software used for data analysis was GC Solution 2.3 and the retention time of 2AP was 14.8 min [30].

### 2.5. Cooked rice elongation percentage determining

The elongation of cooked rice was represented as the difference between the length of the grains measured before and after cooking. In this kind of investigation, precision is therefore very crucial; a MicrotekScanMaker i800 plus scanner was used to perform all length measurements. For each replicate, 10 grains of ripe milled rice were measured using the scanner mentioned above. Each grain was then transferred to a PCR tube containing 150 μL of distilled water. The PCR plate containing the rice grains was then placed in a PCR thermal cycler and the rice grains were individually cooked for 30 min at a block temperature of 99°C. The cooked rice grains were then removed from the plate PCR and placed on filter paper. After drying for 5 min at room temperature, they were re-measured. As with the raw grains, 10 cooked grains were measured again simultaneously; this made it possible to control the variability of the water content which could have resulted from unequal standing times between measurements.

In other to determine the percent elongation of cooked rice for each treatment, the following formula was used:

$$\%\text{E}=\frac{\left(\text{ACML-BCML}\right)}{\text{BCML}}\times 100$$

%E is Elongation percentage; ACML is After Cooking Mean Length and BCML is Before Cooking Mean Length [30].

### 2.6. Amylose content percentage

To determine the amylose content percentage, we have used FOSS INFRATEC^TM^ 1241 ANALYSER (FOSS Nils DK-3400 Hilleroed Denmark) Part no: 10014925 with a temperature range of 0–42°C regulated by the machine. 250 g of milled rice sample from each treatment was used in three replicates to determine amylose content, which is immediately read after each operation.

### 2.7. Fragrance related enzymes and related compounds determination

#### 2.7.1- Proline

The proline contents were estimated according to [31]. 0.3 g grains of each treatment were homogenized in 5 mL of 3% sulfosalicylic acid, boiled for 10 min in water then cooled down. 2 mL of the filtrate was mixed with ninhydrin reagent (3 mL) and glacial acetic acid (2 mL). The mixture was again placed in boiling water for 30 min and then cooled down in an ice bath before been extracted with 4 mL of toluene. The mixture was centrifuged at 4000 × *g* for 5 min. The toluene extraction and the absorbance of the red chromophore were measured at 520 nm and the proline contents were estimated by comparing with a standard curve and expressed as microgram per gram (μg g^-1^ fresh weight (FW)).

#### 2.7.2- Proline dehydrogenase (PDH)

The determination of proline dehydrogenase (PDH) activity was based on the method of [32], and the unit of enzyme activity was U g^-1^ FW (measured as fresh mass). Proline dehydrogenase (PDH) (EC 1.5.99.8) was extracted from fresh leaves and grains (1 g) by homogenization in 2 mL of cooled potassium phosphate buffer (0.1 M, pH 7.8) containing 0.5% (v/v) of Triton X-100 and 1% (w/v) of insoluble polyvinylpolypyrrolidone (PVPP). The resulting suspension was filtered through two layers of cheesecloth and the filtrate was centrifuged for 20 min at 10,000 × *g* at 4°C. The supernatants were desalted on a Sephadex G-25 column (Pharmacia AB, Sweden) and eluted with 0.05 M Tris HCl buffer (pH 7.4) containing 10% glycerol. The extracts were used immediately for the test. To determine PDH activity, the reaction mixture contained L-proline (15 mM), cytochrome c (0.01 mM), phosphate buffer (0.1 M, pH 7.4), 0.5% (v/v) of Triton X-100 and an enzyme extract (0.2 mL) in a total volume of 1ml was used. The reaction mixture was incubated at 37°C for 30 min and the reaction was stopped by adding 1 ml of 10% trichloroacetic acid (TCA). Absorbance was measured at 440 nm.

#### 2.7.3- Δ 1pyrroline-5-carboxylate synthetase (P5CS)

Δ 1-pyrroline-5-carboxylate synthetase (P5CS) activity was determined by referring to the method of [33,34]. The reaction solutions contained 10 mM ATP, 20.0 mM MgCl_2_, 50 mM Tris–HCl buffer, 50 mM sodium glutamate, 100 mM hydroxamate-HCL and 0.5 mL of enzyme extract. The prepared mixture was kept in a 37°C water bath for 5 min, and then the reaction was terminated by the addition of 0.5 mL of a stop buffer (2.5% FeCl 3 and 6% TCA, dissolved in 100 ml of 2.5 M HCl). The P5CS activity was expressed as U g^-1^ FW. The absorbance was measured at 535 nm.

**2.7.4- Δ 1-Pyrroline-5-carboxylate (P5C).** The P5C concentration was determined according to a previously described method [35]. Fresh leaves or rice seed (100 mg) were ground into powder with liquid nitrogen and resuspended in 375 μL of extraction solution containing 50 mM Tris-HCl (pH 8.0), 10% glycerol, 1% triton X-100, and 1% β-mercaptoethanol. The mixture was votexed for 1 min and kept in an ice bath for 2 min. The above procedure was repeated 5 times and then the mixture was allowed to stand for 30 min. After centrifugation at 14000 × *g* for 30 min at 4°C, the supernatant was added to a mixture containing 500 μL of 10% trichloroacetic acid and 125 μL of 40 mM γ-aminobenzaldehyde. The absorbance was measured at 440 nm after the reaction, and the P5C concentration was expressed as μmol g^-1^ FW.

#### 2.7.5- Methylglyoxal (MG)

Methylglyoxal (MG) content in leaf and grain samples was measured according to the method originally developed by [35] in μmol g^-1^ FW. Rice tissues (leaves and grains) (500 mg) were ground to fine powder in liquid nitrogen and resuspended in 5 mL of 0.5 N perchloric acid (PCA; Sigma-Aldrich). The mixture was homogenized with a sonicator for 5 min and then centrifuged at 8000 × *g* at 4°C for 20 min. The supernatant was neutralized with saturated potassium carbonate solution and kept at room temperature for 15 min. Activated charcoal was added to remove the pigments. After centrifugation (8000 × *g* for 10 min), the supernatant (1 mL) was collected and mixed with 200 μL of 7.2 mM 1,2-diaminobenzene (derivatizing agent; Sigma-Aldrich), 100 μL of 10 mM 2,3-dimethyl quinoxaline (internal standard; Sigma-Aldrich), 200 μL of 5 M PCA, and 500 μL of deionized water. After incubation at 4°C for 24 h, the sample was passed through a Sep-Pak C18 Cartridge (Waters, Milford, Mass., U.S.A.), which was pretreated with 8 mL of acetonitrile and 8 mL of 10 mM KH2PO4 (pH 2.5). After washing with 2 mL of 10 mM KH2PO4 (pH 2.5), the cartridge was eluted with 1.5 mL of acetonitrile. The eluates were subjected to HPLC analysis. The quinoxaline derivative of MG and the quinoxaline internal standard (5-MQ) were measured using a Hitachi HPLC system (L-6200A, Tokyo, Japan) equipped with a Mightysil-C18 column (4.6 × 250 mm, 5 μm particles Kanto Chemicals, Tokyo, Japan) and a UV detector at 320 nm. The mobile phase employed was a 32/68 (by volume) mixture of acetonitrile/KH2PO4 (10mM, pH 2.5) at a flow rate of 1 mL/min and the injection volume was 20 μL.

### 2.8. Statistical analysis

The data collected from the experiment were analyzed with Microsoft Excel 2017 (Microsoft, Redmond, WA, USA). Analysis of variance (two ways ANOVA) and correlation coefficients were performed using Statistix version 8 (Analytical, Tallahassee, Florida, USA). The differences among means were separated by using the least significant difference (LSD) test at 5% significance level. Differences in lowercase letter (in the figures and tables), indicate significant difference among the mean values.

## 3. Results

### 3.1. Effects of different sources of N on tillering

**Fig 1:**
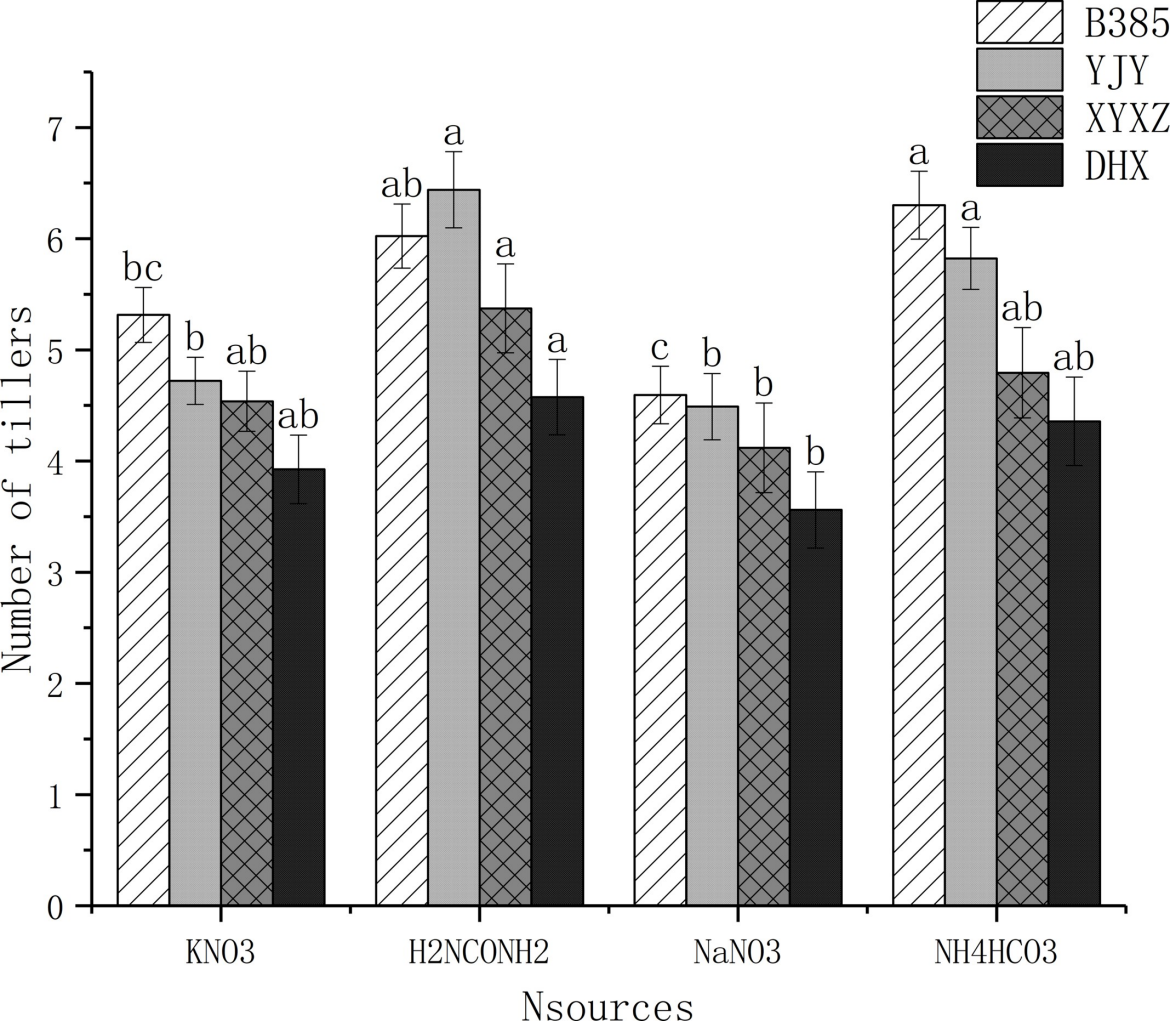
Effect of N sources on rice tillers. Values sharing a common letter do not differ significantly at P < 0.05 level within a variety.

There were some differences among different treatment regarding number of tillers across all the four rice Varieties “Fig 1”. For B385, the highest number of tillers was recorded in NH_4_HCO_3_ treatment (6.30) while the NaNO_3_ treatment (4.59) had the lowest number of tillers. For YJY, the highest number of tillers was recorded in H_2_NCONH_2_ treatment (6.44), followed by NH_4_HCO_3_ treatment (5.82) while the KNO_3_ treatment (4.22) had the lowest number of tillers. For XYXZ, the highest number of tillers was recorded in H_2_NCONH_2_ treatment (5.37) while the NaNO_3_ treatment (4.11) reported the lowest number of tillers. For DHX, the highest number of tillers was recorded in H_2_NCONH_2_ treatment (4.75), followed by NH_4_HCO_3_ treatment (4.35) while the NaNO_3_ treatment (3.56) had the lowest number of tillers. Overall, compared with KNO_3_ and NaNO_3_ treatments, H_2_NCONH_2_ treatment significantly improved the average number of tillers across all the four rice Varieties “Fig 1”.

### 3.2. Effects of nitrogen sources on yield

The grain yield and its components varied significantly with different N sources applications for all the four rice Varieties “Table 1”. The highest grain yield pot^-1^ and number of panicles pot^-1^ were both recorded under NH4HCO3 treatment in all the four varieties compared to other N treatments. The Mean grain yields pot^-1^ of B385, YJY, XYXZ and DHX under NH4HCO3 were 30.74 g, 27.19 g, 39.70 g and 31.20 g, respectively, while the mean for number of panicles pot^-1^ were 31.72, 28.30, 21.28 and 22.80, respectively under the same treatment. Regarding 1000-grain-weight, KNO_3_ produced the highest means in all the four varieties compare to other N sources with mean values of 18.92 g, 24.25 g, 21.64 g and 26.52 g, respectively.

**Table 1:** The effect of Nitrogen sources on yield.

Nitrogen sources	B385			YJY			XYXZ			DHX		
	Number of paniclespot^-1^	1000-Grain weight (g)	Yield pot^-1^ (g)	Number of paniclespot^-1^	1000-Grain weight (g)	Yield pot^-1^ (g)	Number of paniclespot^-1^	1000-Grain weight (g)	Yield pot^-1^ (g)	Number of paniclespot^-1^	1000-Grain weight (g)	Yield pot^-1^ (g)
**KNO**_**3**_	20.47 b	18.92 a	20.79 c	27.86 b	24.25 a	20.18 c	20.80 b	21.64 a	27.37 c	22.29 a	26.52 a	21.31 c
**H**_**2**_**NCONH**_**2**_	20.54 b	18.72 b	30.32 ab	27.20 b	22.65 c	22.28 b	20.66 b	20.39 b	33.11 b	21.67 b	25.95 b	26.35 b
**NaNO**_**3**_	18.17 c	18.57 b	21.37 b	25.05 c	23.07 b	20.71 c	19.51 c	20.19 b	31.95 b	21.03 b	25.90 b	21.41 c
**NH**_**4**_**HCO**_**3**_	31.72 a	18.07 c	30.74 a	28.30 a	22.80 c	27.19 a	21.28 a	19.39 c	39.70 a	22.80 a	23.64 c	31.20 a
**Mean**	19.23*	18.57*	25.81*	27.10*	23.44*	22.59*	20.56*	20.40**	33.03*	21.94*	25.50**	25.06*

*Numbers with the same letter, do not differ significantly at P < 0.05 level within a variety. Means of the four rice cultivars followed by asterisk(s) (*, **) differ significantly at P < 0.05 and P < 0.01, respectively.

### 3.3. 2AP content (μg kg^-1^) in the leaves and grains of different cultivars as affected by nitrogen sources at heading stage and maturity

The effect of N sources on 2AP accumulation in the leaves and the grains of rice is shown in “Table 2”. Different treatment significantly influenced the leaves and grains of 2AP concentration of fragrant rice cultivars. In this study, we observed significant differences in all the cultivars grown with different N sources. 2AP content in the leaves tissues at the heading stage showed significant differences for B385, YJY, XYXZ and DHX rice cultivars. NH_4_HCO_3_ has the highest 2AP content (285.64 μg kg^-1^, 220.87 μg kg^-1^, 282.98 μg kg^-1^ and 275.41 μg kg^-1^, respectively) followed by H_2_NCONH_2_ (276.67 μg kg^-1^, 199.81 μg kg^-1^, 277.78 μg kg^-1^ and 262.54 μg kg^-1^, respectively) while the lowest 2AP content was observed under KNO_3_ treatment. Similarly, 2AP contents in leaves at the maturity stage showed significant differences for each cultivar and NH_4_HCO_3_ had the highest 2AP content (166.36 μg kg^-1^, 130.84 μg kg^-1^, 189.12 μg kg^-1^ and 141.22 μg kg^-1^, respectively) and the lowest 2AP content was observed under NaNO_3_ for each cultivar. Also, 2AP contents in grains at the maturity stage showed significant differences for each cultivar and NH_4_HCO_3_ had the highest 2AP content (87.87 μg kg^-1^, 111.91 μg kg^-1^, 74.24 μg kg^-1^, 117.35 μg kg^-1^ for B385, YJY, XYXZ and DHX rice cultivars, respectively) while the lowest 2AP content was reported under NaNO_3_.

**Table 2:** 2AP content (μg kg^-1^) in the leaves and grains of different cultivars.

Nitrogen sources	B385	YJY	XYXZ	DHX
**Heading stage leaves**				
KNO_3_	153.72 ± 1.92 d	111.69 ± 8.82 cd	143.22 ± 2.89 d	202.07 ± 2.06 d
H_2_NCONH_2_	276.67 ± 6.77 ab	199.81 ± 2.85 b	277.78 ± 7.52 ab	262.54 ± 6.56 b
NaNO_3_	199.81 ± 4.21 c	116.65 ± 1.24 c	192.40 ± 8.20 c	222.99 ± 4.23 c
NH_4_HCO_3_	285.64 ± 8.62 a	220.87 ± 8.15 a	282.98 ± 1.56 a	275.41 ± 8.86 a
Mean	228.96*	162.25*	224.09*	240.76*
		**Matured leaves**		
KNO_3_	144.98 ± 0.36 c	107.04 ± 0.04 c	129.67 ± 0.39 c	127.65 ± 0.09 c
H_2_NCONH_2_	156.77 ± 0.27 b	122.26 ± 0.25 ba	160.84 ± 0.20 b	131.95 ± 0.08 b
NaNO_3_	135.80 ± 0.25d c	105.65 ± 0.13 dc	107.12 ± 0.94 d	103.08 ± 0.11 d
NH_4_HCO_3_	166.36 ± 0.13 a	130.84 ± 0.19 a	189.12 ± 0.35 a	141.22 ± 0.27 a
Mean	150.98	116.45*	146.69*	125.98*
		**Grains**		
KNO_3_	66.40 ± 0.96 cb	96.15 ± 0.33 c	66.78 ± 0.13 c	98.44 ± 0.18 c
H_2_NCONH_2_	69.69 ± 0.68 b	105.91 ± 0.17 ab	70.71 ± 0.36 ab	108.41 ± 0.20 ab
NaNO_3_	47.53 ± 0.15 d	84.51 ± 0.59 d	58.44 ± 0.19 dc	86.50 ± 0.23 d
NH_4_HCO_3_	87.87 ± 0.14 a	111.91 ± 0.14 a	74.24 ± 0.30 a	117.35 ± 0.50 a
Mean	67.89**	99.62**	67.55**	102.68**

Values sharing a common letter within the same variety do not differ significantly at P < 0.05 level. Means of the four rice cultivars followed by asterisk(s) (*, **) differ significantly at P < 0.05 and P < 0.01 level within the same variety respectively.

### 3.4. Cooked rice elongation percentage

**Fig 2:**
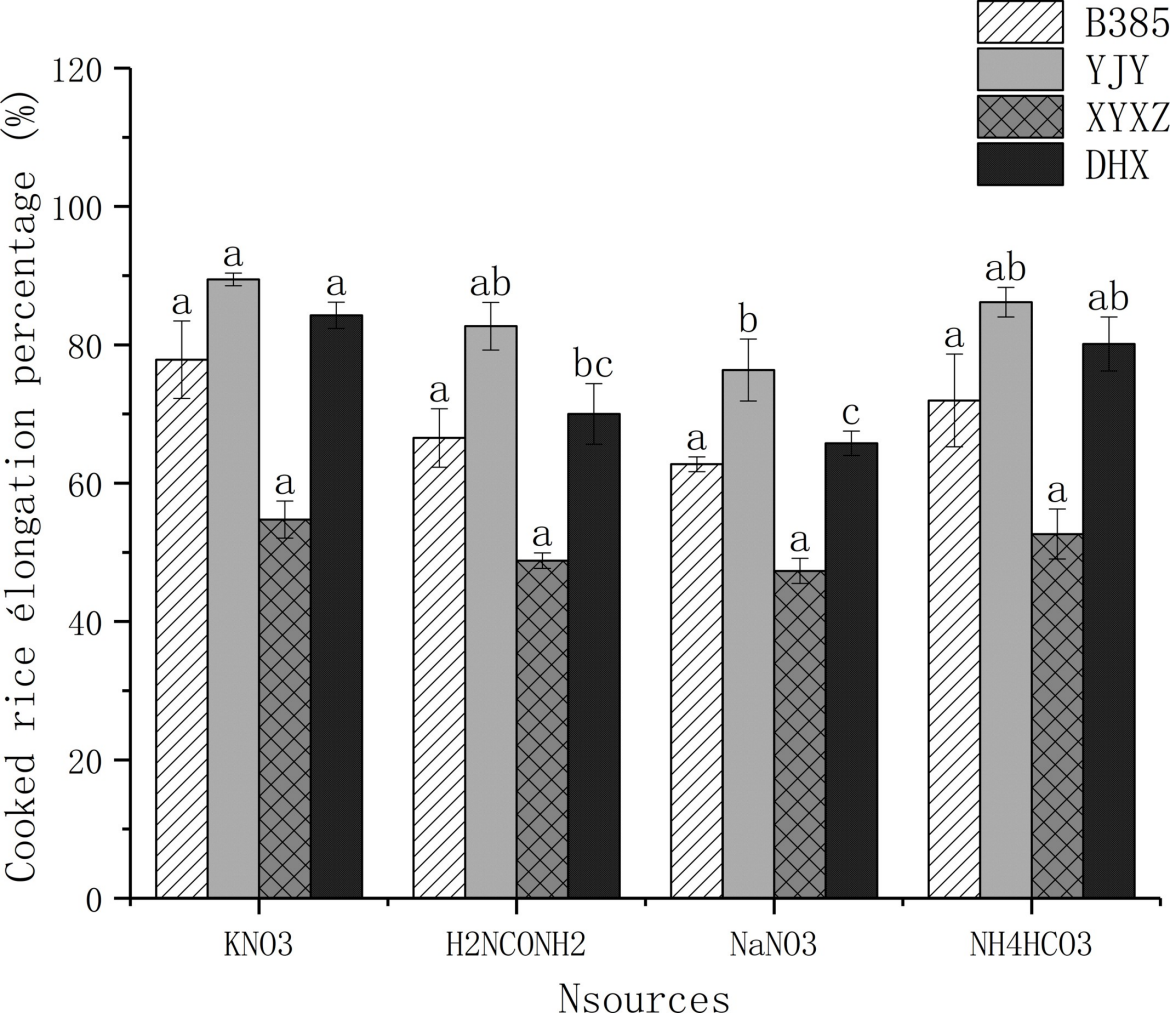
Nitrogen sources effect on cooked rice elongation percentage. Values sharing a common letter for the same variety do not differ significantly at P < 0.05 according to LSD test.

“Fig 2” shows the cooked rice elongation percentage of B385, YJY, XYXZ and DHX grown in the different N sources. Here, we reported that the cooked rice elongation percentage of B385 harvested from rice plants that were grown in KNO_3_, H_2_NCONH_2_, NaNO_3_ and NH_4_HCO_3_ regimes were 77.85%, 66.54%, 62.74% and 71.96%, respectively. In addition, the cooked rice elongation percentage of YJY grown in KNO_3_, H_2_NCONH_2_, NaNO_3_ and NH_4_HCO_3_, were 89.46%, 82.69%, 76.35%, and 86.18%, respectively. Furthermore, the cooked rice elongation percentage of XYXZ harvested from rice plants grown in KNO_3_, H_2_NCONH_2_, NaNO_3_ and NH_4_HCO_3_ nitrogen regimes were 54.73%, 48.80%, 47.32% and 52.65%, respectively. For DHX, results of the cooked rice elongation percentage of harvested rice plant grown in KNO_3_, H_2_NCONH_2_, NaNO_3_ and NH_4_HCO_3_ regimes were 84.28%, 70%, 65.77% and 80.13%, respectively. Overall, we also observed that the grains harvested from rice plant that were grown in KNO_3_ treatment had the highest cooked rice elongation percentage when compared to other nitrogen source regimes.

### 3.5. Effect of different sources of nitrogen on proline content, P5C and P5CS activities

Nitrogen application significantly improved P5C, proline content and P5CS activities of enzymes involved in 2AP biosynthesis. For instance in P5C, significant improvements in leaves at the onset of heading were 1.75 μmol g^-1^, 1.67 μmol g^-1^, 1.71 μmol g^-1^ and 1.48 μmol g^-1^ in B385, YJY, XYXZ and DHX, respectively. For leaves at maturity, 1.33 μmol g^-1^, 1.24 μmol g^-1^, 1.49 μmol g^-1^ and 1.55 μmol g^-1^ were observed in B385, YJY, XYXZ and DHX, respectively. In addition, 0.38 μmol g^-1^, 0.31 μmol g^-1^, 0.30 μmol g^-1^ and 0.36 μmol g^-1^ in matured grains were reported in B385, YJY, XYXZ and DHX, respectively under NH_4_HCO_3_ treatment compared to the other nitrogen sources.

Similarly, Proline content also showed significant improvements towards exogenous feeding of nitrogen sources where maximum values for leaves at heading (41.21 μg g^-1^, 41.94 μg g^-1^, 40.77 μg g^-1^ and 38.38 μg g^-1^), in leaves at maturity (35.06 μg g^-1^, 39.75 μg g-1, 42.02 μg g^-1^ and 38.13 μg g^-1^), in matured grains (13.71μg g^-1^, 13.19 μg g^-1^, 13.80 μg g^-1^ and 12.44 μg g^-1^), were recorded in B385, YJY, XYXZ and DHX, respectively.

Additional, KNO_3_, H_2_NCONH_2_, NaNO_3_ and NH_4_HCO_3_ treatments differentially affected the activities of P5CS in all the four rice cultivars. Compared with all the treatments, the activities of P5CS were higher in B385 rice cultivar (37.44 U g^-1^ FW) under NaNO_3_ treatment in leaves at the heading stage. In the leaves at maturity and matured grains, the activities of P5CS were found higher in YJY and DHX rice cultivars (46.29 U g^-1^ FW and 2.88 U g^-1^ FW) under H_2_NCONH_2_ treatment. Statistically, no significant difference was noted among the treatments for P5CS activity in both at heading and matured leaf stages in DHX rice cultivar for all the treatments “Table 3”. Overall, H_2_NCONH_2_ and NH_4_HCO_3_ treatments accumulated more P5C, proline and P5CS activities than KNO_3_ and NaNO_3_ treatments across the four cultivars “Table 3”.

**Table 3:** Effect of different sources of nitrogen on P5CS activity, P5C activity, and proline content.

Treatments	P5C activity (μmol g^-1^)			Proline content (μg g^-1^)			P5CS activity (U g^-1^ FW)		
	Leaves		Grains	Leaves		Grains	Leaves		Grains
	Heading	Matured	Matured	Heading	Matured	Matured	Heading	Matured	Matured
**Basmati 385**									
**KNO**_**3**_	1.45 d	1.25 c	0.29 c	29.43 d	26.20 c	5.37 d	31.01 c	38.61 a	2.29 bc
**H**_**2**_**NCONH**_**2**_	1.68 c	1.36 a	0.34 ab	38.64 b	35.06 a	12.76 ab	29.97 c	35.38 b	2.68 a
**NaNO**_**3**_	1.72a b	1.25 c	0.29 c	33.29 c	31.08 ab	8.82 c	37.44 a	36.12 c	2.37 c
**NH**_**4**_**HCO**_**3**_	1.75 a	1.33 ab	0.38 a	41.21 a	30.59 ab	13.71 a	33.72 ab	36.30 ab	2.64 ab
**Mean**	1.65*	1.29*	0.30*	35.64*	30.73**	8.14*	32.93*	36.60*	2.38**
***Yunjingyou YJY***									
**KNO**_**3**_	1.57 ab	1.19 c	0.30 a	41.94 a	31.99 c	6.09 c	26.63 ab	33.87 c	2.28 c
**H**_**2**_**NCONH**_**2**_	1.45 b	1.28 a	0.29 ab	35.72 b	39.75 a	11.03 ab	26.66 ab	46.29 a	2.68 a
**NaNO**_**3**_	1.43 b	1.05 c	0.28 b	32.63 b	36.28 ab	7.51 b	29.20 c	38.05 b	2.28 c
**NH**_**4**_**HCO**_**3**_	1.67 a	1.24 ab	0.31 a	30.33 b	27.28 dc	13.19 a	29.22 a	36.82b c	2.34 ab
**Mean**	1.56*	1.19**	0.29*	35.15*	33.82*	9.45*	27.92*	38.75*	2.39**
**Xiangyaxiangzhan XYXZ**									
**KNO**_**3**_	1.57 bc	1.29 c	0.27 c	40.77 a	36.13 b	7.44 c	32.01 a	35.84 b	2.46 b
**H**_**2**_**NCONH**_**2**_	1.82 a	1,15 d	0.29 ab	39.45 ab	33.82 cb	12.33 ab	28.82 c	37.33 a	2.74 a
**NaNO**_**3**_	1.68 b	1.48 a	0.27 c	32.76 b	30.02 d	9.95 b	30.79 b	35.74 b	2.37 c
**NH**_**4**_**HCO**_**3**_	1.71 ab	1.49 a	0.30 a	28.63 cb	42.02 a	13.80 a	31.64 ab	36.89 a	2.25 c
**Mean**	1.69*	1.35*	0.28**	35.40**	34.97*	10.39*	30.81*	36.45*	2.45*
***Daohuaxiang DHX***									
**KNO**_**3**_	1.44 a	1.34 b	0.30 b	38.38 a	35.06 ab	8.68 cb	31.03 a	37.64 a	2.58 b
**H**_**2**_**NCONH**_**2**_	1.33 b	1.36 b	0.33 ab	36.54 a	29.74 c	12.22 a	30.84 a	38.87 a	2.88 a
**NaNO**_**3**_	1.36 b	1.53 ab	0.29 b	30.57 b	27.41 c	9.16 b	31.56 a	38.58 a	2.38 c
**NH**_**4**_**HCO**_**3**_	1.48 a	1.55 a	0.36 ab	21.71 c	38.13 a	12.44 a	29.28 a	37.19 a	2.79 ab
**Mean**	1.40*	1.44**	0.33**	31.85*	32.58*	10.62**	30.17*	38.07*	2.50**

Values sharing a common letter within a column do not differ significantly at P < 0.05. Means of the four rice cultivars followed by asterisk(s) (*, **) differ significantly at P < 0.05 and P < 0.01 level within the same variety.

### 3.6. Effect of nitrogen sources treatments on pyrroline content, PDH and Methylglyoxal (MG) activities

As shown in “Table 4”, the PDH, pyrroline contents and Methylglyoxal (MG) activities were significantly affected by different N sources. For PDH, at the onset of heading leaves, matured leaves and grains, the PDH activity in NH_4_HCO_3_ treatment were significantly higher than KNO_3_, H_2_NCONH_2_, and NaNO_3_ treatments for B385, YJY, XYXZ and DHX rice cultivars, respectively with the exception of KNO_3_ under B385 and NaNO_3_ under DHX cultivars in matured leaves, which were higher than NH_4_HCO_3_ treatment. For Pyrroline content, at the onset of heading leaves, matured leaves and grains the highest activities were recorded in NH_4_HCO_3_ treatment and the lowest activities were recorded in NaNO_3_ treatment across the four cultivars with the exception of KNO_3_ and NaNO_3_ under DHX cultivars, which were significantly lower than NaNO_3_ treatment at the onset of heading leaves.

**Table 4:** Effect of different sources of Nitrogen on PDH activity, Pyrroline content, and Methylglyoxal (MG) activity.

Treatments	PDH activity (μmol g^-1^)			Pyrroline content (μg g^-1^)			MG activity (U g^-1^ FW)		
	Leaves		Grains	Leaves		Grains	Leaves		Grains
	Heading	Matured	Matured	Heading	Matured	Matured	Heading	Matured	Matured
**Basmati 385**									
**KNO**_**3**_	18.43 b	15.67 a	24.43 b	9.81 b	17.18 a	5.06 a	203.83 a	122.46 a	61.23 c
**H**_**2**_**NCONH**_**2**_	19.60 b	14.02 b	25.53 ab	8.22 c	14.55 b	4.97 ab	205.26 a	124.20 a	62.10 c
**NaNO**_**3**_	22.70 a	13.47 c	23.57 b	9.24 b	11.73 c	4.97 ab	201.46 a	22.43 b	94.55 b
**NH**_**4**_**HCO**_**3**_	25.28 a	14.31 b	27.05 a	10.52 a	14.37 b	5.18 a	210.46 a	22.26 b	111.33 a
**Mean**	21.50*	14.36*	25.14*	9.44*	14.45*	5.04*	205.25*	72.83*	82.30**
***Yunjingyou* YJY**									
**KNO**_**3**_	19.13 c	17.88 cb	27.93 c	7.70 b	7.12 c	4.96b a	169.3 b	165.2 bc	82.60 b
**H**_**2**_**NCONH**_**2**_	17.17 d	18.56 b	32.48 ab	7.60 b	7.91 ab	5.08 ab	166.83 b	170.06 b	85.03 b
**NaNO**_**3**_	21.37 b	15.96 c	31.30 ab	7.80 b	8.13 a	4.87 b	160.53 bc	194.73 a	92.36 ab
**NH**_**4**_**HCO**_**3**_	27.17 a	25.35 a	34.37 a	8.30 a	7.77 ab	5.16 a	173.80 a	184.9 ab	97.45 a
**Mean**	21.15*	19.43*	31.52*	7.85*	7.73*	5.01*	167.61*	178.72*	89.36*
**Xiangyaxiangzhan XYXZ**									
**KNO**_**3**_	27.80 ab	18.05 ab	31.59 b	8.71 ab	8.13 a	5.01 a	188.53 ab	175.9 b	87.95 b
**H**_**2**_**NCONH**_**2**_	18.43 c	18.59 ab	32.53 ab	8.03 ab	7.96 b	4.96 ab	155.53 ab	176.63 b	88.31 b
**NaNO**_**3**_	20.85 b	17.74 cb	31.05 b	8.84 ab	7.94 b	4.94 ab	186.43 b	203.40 a	86.56 b
**NH**_**4**_**HCO**_**3**_	28.26 a	19.61 a	34.32 a	9.18 a	8.15 a	5.23 a	211.03 a	173.13 b	101.70 a
**Mean**	23.83*	18.49*	32.37*	8.69*	8.04*	5.03*	185.38*	182.26*	91.13*
***Daohuaxiang* DHX**									
**KNO**_**3**_	28.48 b	17.69 cb	30.96 b	9.05 a	7.94 cb	5.01 b	181.63 b	192.76 bc	96.38 b
**H**_**2**_**NCONH**_**2**_	17.41 cb	18.27 b	31.98 b	8.96 b	9.57 a	5.04 b	183.93 ab	193.10 b	96.55 b
**NaNO**_**3**_	28.02 b	19.21 a	32.62 a	7.70 c	8.29 b	5.01 b	186.43 a	207.30 a	91.050 cb
**NH**_**4**_**HCO**_**3**_	30.13 a	18.85 ab	33.93 a	9.17 a	9.26 a	5.21 a	182.34 ab	182.10 c	103.65 a
**Mean**	26.01*	18.50*	32.37*	8.72*	8.76*	5.06**	183.58**	193.81*	96.90*

Values sharing a common letter within a column do not differ significantly at P < 0.05. Means of the four rice cultivars followed by asterisk(s) (*, **) differ significantly at P < 0.05 and P < 0.01 level within the same variety.

Furthermore, KNO_3_, H_2_NCONH_2_, NaNO_3_ and NH_4_HCO_3_ treatments significantly affected the activities of Methylglyoxal (MG) activities in all the four rice cultivars. Compared with all the treatments, the activities of MG were higher in XYXZ rice cultivar (211.03 U g^-1^ FW) under NH_4_HCO_3_ treatment in leaves at the heading stage. In the leaves at maturity, the activities of MG were higher in DHX rice cultivar (207.30 U g^-1^ FW) under NaNO_3_ treatment. In matured grains, the activities of MG were found higher in DHX rice cultivar (111.33 U g^-1^ FW) under NH_4_HCO_3_ treatment. Statistically, no significant difference noted among the treatments for MG activities at the onset of heading leaves in DHX rice cultivar for all the treatments “Table 4”. Overall, NH_4_HCO_3_ treatments accumulated more PDH, pyrroline contents and Methylglyoxal (MG) activities than KNO_3,_ H_2_NCONH_2_ and NaNO_3_ treatments across the four cultivars “Table 4”.

### 3.7. Amylose content percentage

**Fig 3:**
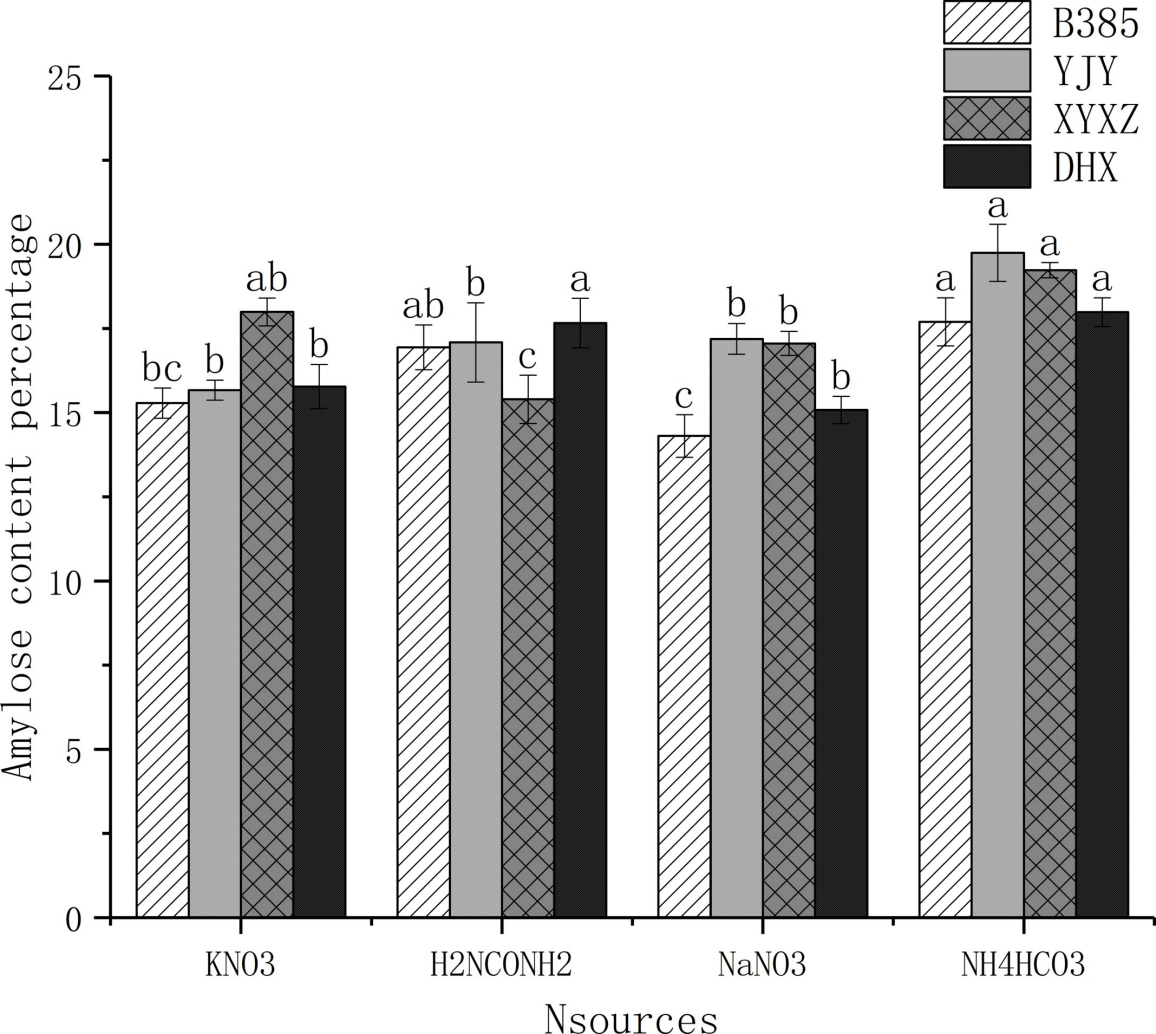
Amylose content percentage for B385, YJY, and XYXZ and DHX rice cultivars. Values sharing a common lower-case letter for the same variety do not differ significantly at P < 0.05 according to LSD test.

Results showed the amylose content percentage of B385, YJY, XYXZ and DHX grown in different N sources “Fig 3”. The amylose content percentage of B385 harvested from rice plants that were grown in KNO_3_, H_2_NCONH_2_, NaNO_3_ and NH_4_HCO_3_ regimes were 15.30%, 16.95%, 14.31% and 17.70% respectively. Also, the amylose content percentage of YJY grown in KNO_3_, H_2_NCONH_2_, NaNO_3_ and NH_4_HCO_3_, were 15.68%, 17.09%, 17.20%, and 19.76%, respectively. Amylose content percentage of XYXZ grown in KNO_3_, H_2_NCONH_2_, NaNO_3_ and NH_4_HCO_3_, were 18.0%, 15.40%, 17.06%, and 19.23%, respectively. Furthermore, the amylose content in the grains of DHX harvested from rice plants that were grown in KNO_3_, H_2_NCONH_2_, NaNO_3_ and NH_4_HCO_3_ regimes were 15.68%, 17.09%, 17.20%, and 19.76%, respectively. Additionally, we also observed that the grains harvested from rice plant that were grown in NH_4_HCO_3_ treatment recorded the highest amylose content percentage when compared to other nitrogen source regimes “Fig 3”.

## 4. Discussion

Many studies have shown that nitrogen affects rice in various ways, for instance [36] demonstrated that, application of nitrogen at the booting stage affects the 2-acetyl-1-pyrroline, proline and total nitrogen contents of aromatic rice; Different rice genotypes differed in their rice growth, development and grain yield in response to different nitrogen application rates and nitrogen types [37–43]. In this study, significant differences in grain yield and yield related traits were observed for cultivars and nitrogen applications. For number of tillers, our findings revealed that each cultivar was significantly affected by nitrogen sources and H_2_NCONH_2_ treatment recorded highest average number of tillers across all the four rice Varieties “Fig 1”. Nonetheless [44,45], reported that nitrogen source from urea increased the number of productive tillers in rice. In addition, growth and yield attributes like number of panicles and 1000-grains weight were segregated under different nitrogen source, according to the cultivars “Table 1”. Besides, in this study, nitrogen application treatment of NH_4_HCO_3_ resulted in higher number of paniclespot^-1^ and grain yieldpot^-1^ than KNO_3_, H_2_NCONH_2_ and NaNO_3_ treatments. Also, there is a significant difference between nitrogen sources for the 1000-grain weight and KNO_3_ showed the best result in each rice cultivar: 18.92 g, 24.25 g, 21.64 g, and 26.52 g for B385, YJY, XYXZ and DHX respectively. Previous studies have shown that the number of spikelets per panicle, percentage grain filling and grain weight, were all affected by different nitrogen fertilizer [46,47]. Similarly [20], demonstrated that, Potassium source applied as panicle fertilizer significantly increases the grain yield and quality by helping in translocation of photosynthetic products and other plant metabolites, thus contributing to improved grain quality. It has also been reported that higher K rates result in a stronger aroma, whiter and glassier appearance and lower softness in basmati rice [48]. Overall, the general positive nitrogen effect in the regulating of fragrant rice genotypes had also been assessed.

2AP content (μg kg^-1^) in leaves, grains and related enzymes involved in 2AP biosynthesis affected by nitrogen sources at different stage. In this study, we tried to delineate the relationships between different N sources and 2AP biosynthesis in two parts of the rice plant, including grains at two growth stage. Our results demonstrate that N sources (heading stage) improved the 2AP contents in leaves. 2AP content in the leaves tissues at the heading stage showed significant differences for B385, YJY, XYXZ and DHX rice cultivars and NH_4_HCO_3_ reported the highest 2AP content (285.64 μg kg^-1^, 220.87 μg kg^-1^, 282.98 μg kg^-1^ and 275.41 μg kg^-1^, respectively) “Table 2”. Also, in the matured leaves and grains there were significant differences between treatments. Hence, in grains, the highest 2AP content was observed under NH_4_HCO_3_ (166.36 μg kg^-1^, 130.84 μg kg^-1^, 189.12 μg kg^-1^ and 141.22 μg kg^-1^ for B385, YJY, XYXZ and DHX, respectively). Previous studies have shown that 2AP content varies depending not only on the planting site and environmental conditions but also on the plant, genotype, cultivar and even part of the plant [24,49,50]. Also, knowing the role that potassium plays for nitrogen like ensuring efficient utilization of N [51], the low values obtained in the treatment of KNO_3_ would be due to the need that potassium has only facilitated the assimilation of nitrogen without bringing a significant impact in the increase of 2AP [20]. In addition, nitrogen sources such as urea H_2_NCONH_2_ and NH_4_HCO_3_ present a great advantage for the availability of nitrogen for plant growth and for 2AP biosynthesis [21]. In the present study, H_2_NCONH_2_ and NH_4_HCO_3_ sources increased the 2AP contents in the rice leaves at different stages and grains “Table 2”.This could be explained by the available form of nitrogen for rice plants nutrition [22]. Forms of N in NO_3_^-^ are first converted to NO_2_^-^ then to NH_4_^+^ by the sequential actions of the enzymes Nitrate reductase (NR) and nitrite reductase (NiR), while NH_4_^+^ is directly assimilated into amino acids via the concerted activities of the GS enzymes and GOGAT [52]. Thus, the present study examined the influence of nitrogen sources (H_2_NCONH_2,_ NH_4_HCO_3,_ NaNO_3_ and KNO_3_) application on the biosynthesis of 2AP and in second position the targeted impact of each nitrogen source on different aromatic rice cultivars (B385, YJY, XYXZ and DHX).

Proline content significantly increased (grains and leaves at different stages) Compared with other treatment, the activities of Proline content were higher in NH_4_HCO_3_ fertilizer “Table 3”. Similarly, P5CS and P5C activities were significantly increased in rice grain by NH_4_HCO_3_ fertilizer compared to other nitrogen sources “Table 3”. By the same way, NH_4_HCO_3_ significantly affected 2AP content in the matured grains “Table 2”. Our study showed the role of proline and P5CS activities into the 2AP biosynthesis (pathway) as affected by NH_4_HCO_3_ fertilizer compared to other nitrogen sources. The 2AP content in leaves and grains at different stages revealed a significant correlation with proline content, P5CS and P5C activities. It has been reported that nitrogen management at tillering stage can regulate 2AP accumulation [46,49]. Moreover, it has also been reported that, the 2AP content, proline content, P5C content and P5CS activity were investigated as the important parameters that positively related to the 2AP content in brown rice under nitrogen treatments [53]. 2AP has been detected in different plant tissues of fragrant rice plant such as grain, stem sheath and leaf [9,54,55], in this study we have detected 2AP in leaves at different stages and in grains. Hence, this study showed the advanced impact of NH_4_HCO_3_ on the biosynthesis of 2AP.

PDH is a key enzyme in proline biodegradation pathway and this study showed that the activity of PDH in grains increased with NH_4_HCO_3_ and H_2_NCONH_2_, while the content of 2AP in grains increased significantly compared to other N sources treatments at the same time “Table 3”. Also, Pyrroline and MG increased and following the same trend as Proline content “Table 4”. The correlations between 2AP, enzymes and prediction of 2AP content from different plant tissues and growth stages were assessed according the different nitrogen sources. This study suggested that the enzymes like P5C content, pyrroline, proline content, Methylglyoxal (MG) activity, the P5CS activity and the PDH activity could be found to contribute to 2AP accumulation affected by nitrogen sources and NH_4_HCO_3_ contribute more. Similarly, the enzymes (PDH, P5CS, Methylglyoxal (MG), P5C, Pyrroline and Proline) have been reported to be related to 2AP formation [14,15,26] and other studies revealed some variations in the correlation between the 2AP content and enzymes under different fertilization and varieties [55].

Rice consumers prefer grains with extensive elongation after cooking [28,56] and a high percentage of amylose content [30]. In our study, we observed that each cultivar showed different responses to nitrogen treatment, KNO_3_ and NH_4_HCO_3_ had a higher percentage of elongation of cooked rice “Fig 2”. On the other hand, we notice a higher percentage of amylose in the NH_4_HCO_3_ treatment followed by H_2_NCONH_2_ for almost all the varieties “Fig 3”. This agrees with our results with regards to the high level of the percentage elongation of cooked rice under the treatment of KNO_3_ “Fig 2”. Contrarily, we observed a higher percentage rate of amylose under NH_4_HCO_3_ and H_2_NCONH_2_ “Fig 3” treatments, which could be explained by the rapid and immediate availability of NH_4_^+^ ions compared to other forms of nitrogen for the nutrition of the rice plant [44]. However, information on the effect of different sources of nitrogen on cooked rice elongation percentage and amylose percentage as key quality factors is still very scarce. Our work showed that nitrogen sources such as KNO_3_ increased the percentage elongation of cooked rice; [57] mentioned that Potassium (K) is not easily assimilated into organic matter but helps to improve rice quality.

We further explored the roles of nitrogen sources in improving growth, yield and aromatic characters of fragrant rice. Among different rice cultivars, 2-acetyl-1-pyrroline (2AP) is considered as a principle aroma compound contributing to the aroma character in fragrant rice. Our results indicated that nitrogen sources treatments regulated the proline and Pyrroline content as well as the P5C, Methylglyoxal, PDH and P5CS activities in leaves and grains “Tables 3 and 4”. In order to further elucidate the effect of application of different sources of N on the accumulation of 2AP, we found that the effect of application of N differed depending on the plant tissue and the results corroborate those of [24], who reported that the flavor of the grains depends on the N application rate. Here, we confirmed that the increase in the 2AP content of the grains would depend on different sources of N “Table 5”. Brown rice flavor was found to be directly correlated with total N and proline content [18]. According to the correlation analyses between 2AP biosynthesis, proline content and nitrogen sources, we found that some nitrogen sources (NH_4_HCO_3_ and H_2_NCONH_2_) increased proline content of grains than other nitrogen sources (KNO_3_ and NaNO_3_) “Table 3”. In addition, the content of 2AP in the grains was positively correlated with the content of proline and nitrogen sources such as NH_4_HCO_3_ and H_2_NCONH_2_ in both parts of the plant at the heading stage and at the maturity “Table 5”. Overall, NH_4_HCO_3_ and H_2_NCONH_2_ nitrogen sources increased the proline content, and 2AP content at heading stage and maturity both in leaves and grains, and consequently improved the aroma “Table 4”. Therefore, the application of these different nitrogen fertilizer sources differed in the increased levels of proline in leaves at the heading stage and leaves at the maturity, which ultimately increased the level of 2AP in the grains.

**Table 5:** Correlation analyses between 2AP biosynthesis, proline content and nitrogen sources.

Treatments	2AP content			Proline content (μg g^-1^)		
	Leaves		Grains	Leaves		Grains
	Heading	Matured	Matured	Heading	Matured	Matured
**Basmati 385**						
**KNO**_**3**_	-0.7654	-0.5626	-0.4396	-0.3372	-0.5424	-0.5220
**H**_**2**_**NCONH**_**2**_	-0.6821	-0.7025*	-0.8078*	-0.9273**	-0.8825**	-0.8187*
**NaNO**_**3**_	-0.8515*	-0.4364	-0.2658	-0.5213	-0.7169*	-0.6728
**NH**_**4**_**HCO**_**3**_	-0.9185*	-0.8632*	-0.8975**	-0.7959*	-0.7313*	-0.7398*
***Yunjingyou YJY***						
**KNO**_**3**_	-0.7426*	-0.4925	-0.8822**	-0.8597*	-0.6900	-0.6323
**H**_**2**_**NCONH**_**2**_	-0.7184*	-0.96**	-0.7631*	-0.7078*	-0.9850**	-0.8354*
**NaNO**_**3**_	-0.6441	-0.5664	-0.3741	-0.5558	-0.6020	-0.5728
**NH**_**4**_**HCO**_**3**_	-0.9137**	-0.9222**	-0.7139**	-0.7827*	-0.7233*	-0.7636**
**Xiangyaxiangzhan XYXZ**						
**KNO**_**3**_	-0.4963	-0.4935	-0.7626*	-0.7755*	-0.4428	-0.5968
**H**_**2**_**NCONH**_**2**_	-0.7265*	-0.8694**	-0.7002*	-0.8896*	-0.8271*	-0.7998*
**NaNO**_**3**_	-0.5348	-0.6824	-0.6335	-0.6856	-0.6328	-0.6022
**NH**_**4**_**HCO**_**3**_	-0.7962*	-0.7863*	-0.8022**	-0.8088*	-0.9679**	-0.8955*
***Daohuaxiang DHX***						
**KNO**_**3**_	-0.7822*	-0.6008	-0.5080	-0.6922	-0.5666	-0.3418
**H**_**2**_**NCONH**_**2**_	-0.8321*	-0.8566*	-0.9766**	-0.7700*	-0.8411*	-0.7254*
**NaNO**_**3**_	-0.5478	-0.4239	-0.6408	-0.6814	-0.6875	-0.4322
**NH**_**4**_**HCO**_**3**_	-0.8956*	-0.9636**	-0.8824**	-0.7364*	-0.8729*	-0.8635*

Correlation analysis of 2AP content, Proline content and Nitrogen sources. Correlation at * P < 0.05, **P < 0.01 probability levels.

## 5. Conclusion

In this study, we have demonstrated that the application of different Nitrogen sources had significant effect on growth, yield, amylose content, 2AP and cooked rice elongation percentage. The H_2_NCONH_2_ and NH4HCO3 treatments were found better than KNO_3_ and NaNO_3_ regarding grain yield, rice quality and rice aroma formation. Increased 2AP concentrations might be attributed to higher rates of proline, pyrroline content; P5C, Methylglyoxal, PDH and P5CS activities. The findings reported in the study will be helpful in the manufacture of nitrogen fertilizers that would be used in the production of rice with better grain quality.

## Supporting information

S1 Table Amylose content percentage. (DOCX)

S2 Table Cooked rice elongation (%). (DOCX)

S3 Table Milled rice (mm). (DOCX)

S4 Table 2AP content (μg kg^−1^) in the leaves and grains of different cultivars. (DOCX)
