# Supplementary material for: Nitrogen sources affected the biosynthesis of 2-acetyl-1-pyrroline, cooked rice elongation and amylose content in rice
Source: PLoS One. 2021 Jul 15;16(7):e0254182. doi: 10.1371/journal.pone.0254182 (PMC8282057; doi:10.1371/journal.pone.0254182)
Supplement: S1 Table — (DOCX) [file pone.0254182.s001.docx]

**S1 Table. Amylose content percentage**

| N sources | B385 | YJY | XYXZ | DHX |
| --- | --- | --- | --- | --- |
| KNO3 | 14.52 | 15.08 | 17.53 | 16.82 |
| KNO3 | 15.26 | 16.03 | 18.82 | 15.93 |
| KNO3 | 16.07 | 15.9 | 17.62 | 14.57 |
| H2NCONH2 | 18.2 | 19.05 | 16.5 | 18.56 |
| H2NCONH2 | 16.69 | 17.23 | 15.64 | 18.22 |
| H2NCONH2 | 15.93 | 14.98 | 14.05 | 16.2 |
| NaNO3 | 14.64 | 17.03 | 17.21 | 14.28 |
| NaNO3 | 13.08 | 16.49 | 16.37 | 15.36 |
| NaNO3 | 15.2 | 18.05 | 17.59 | 15.6 |
| NH4HCO3 | 17.6 | 21.4 | 19.67 | 18.7 |
| NH4HCO3 | 16.51 | 19.27 | 18.94 | 18.02 |
| NH4HCO3 | 18.98 | 18.58 | 19.08 | 17.23 |

B385: Basmati 385

YJY: Yunjingyou

XYXZ: Xiangyaxiangzhan

DHX: Daohuaxiang
