# Supplementary material for: Nitrogen sources affected the biosynthesis of 2-acetyl-1-pyrroline, cooked rice elongation and amylose content in rice
Source: PLoS One. 2021 Jul 15;16(7):e0254182. doi: 10.1371/journal.pone.0254182 (PMC8282057; doi:10.1371/journal.pone.0254182)
Supplement: S2 Table — (DOCX) [file pone.0254182.s002.docx]

**S2 Table. Cooked rice elongation (%)**

|  | COOCKED RICE | |  |
| --- | --- | --- | --- |
| B385 | | | |
| H2NCONH2 | KNO3 | NaNO3 | NH4HCO3 |
| 8.147005 | 9.252972 | 8.614267 | 7.997573 |
| 8.177221 | 9.979802 | 8.466681 | 8.99281 |
| 8.103385 | 8.913786 | 7.862284 | 7.897458 |
|  |  |  |  |
|  | DHX | | |
| 9.162196 | 9.967103 | 10.07699 | 9.580342 |
| 8.834355 | 10.2203 | 9.670923 | 10.02943 |
| 9.491627 | 9.982608 | 9.670923 | 9.541571 |
|  |  |  |  |
|  | XYXZ | | |
| 8.704491 | 9.756477 | 9.245046 | 8.835071 |
| 8.765187 | 9.656477 | 9.245046 | 8.234801 |
| 8.2932 | 9.102264 | 8.905721 | 8.742138 |
|  |  |  |  |
|  | YJY | | |
| 7.477915 | 7.918525 | 7.38616 | 7.804966 |
| 7.302032 | 8.093214 | 7.602495 | 7.896448 |
| 7.616494 | 7.823394 | 6.949723 | 7.984772 |

B385: Basmati 385

YJY: Yunjingyou

XYXZ: Xiangyaxiangzhan

DHX: Daohuaxiang
