# Supplementary material for: Nitrogen sources affected the biosynthesis of 2-acetyl-1-pyrroline, cooked rice elongation and amylose content in rice
Source: PLoS One. 2021 Jul 15;16(7):e0254182. doi: 10.1371/journal.pone.0254182 (PMC8282057; doi:10.1371/journal.pone.0254182)
Supplement: S3 Table — (DOCX) [file pone.0254182.s003.docx]

|  | MILLED RICE |  |  |
| --- | --- | --- | --- |
|  |  |  |  |
|  | B385 |  |  |
| H2NCONH2 | KNO3 | NaNO3 | NH4HCO3 |
| 4.706705 | 5.389177 | 5.231195 | 4.740112 |
| 4.870874 | 5.279525 | 5.207922 | 4.86553 |
| 5.107985 | 5.157897 | 4.884327 | 4.86553 |
|  | DHX |  |  |
| 5.295609 | 5.59763 | 5.502758 | 5.21533 |
| 5.474381 | 5.595165 | 5.113533 | 5.440452 |
| 5.404608 | 5.481842 | 5.226628 | 5.536776 |
|  | XYXZ |  |  |
| 5.909745 | 6.225615 | 5.966728 | 6.06691 |
| 5.805801 | 6.109646 | 5.944722 | 5.960515 |
| 6.003898 | 6.091768 | 6.121106 | 6.043599 |
|  | YJY |  |  |
| 4.209442 | 4.24109 | 4.137678 | 4.064514 |
| 4.031313 | 4.311764 | 4.158936 | 4.088271 |
| 4.024017 | 4.162529 | 4.14337 | 4.045771 |

**S3 Table. Milled rice (mm)**

B385: Basmati 385

YJY: Yunjingyou

XYXZ: Xiangyaxiangzhan

DHX: Daohuaxiang
