# Supplementary material for: Nitrogen sources affected the biosynthesis of 2-acetyl-1-pyrroline, cooked rice elongation and amylose content in rice
Source: PLoS One. 2021 Jul 15;16(7):e0254182. doi: 10.1371/journal.pone.0254182 (PMC8282057; doi:10.1371/journal.pone.0254182)
Supplement: S4 Table — (DOCX) [file pone.0254182.s004.docx]

**S4 Table. 2AP content (μg· kg^−1^) in the leaves and grains of different cultivars**

| Nitrogen sources B385 YJY XYXZ DHX | | | | |
| --- | --- | --- | --- | --- |
| **Heading stage leaves** | | | | |
| KNO_3_ | 153.72±1.92d | 111.69±8.82cd | 143.22±2.89d | 202.07±2.06d |
| H_2_NCONH_2_ | 276.67±6.77ab | 199.81±2.85b | 277.78±7.52ab | 262.54±6.56b |
| NaNO_3_ | 199.81±4.21c | 116.65±1.24c | 192.40±8.20c | 222.99±4.23c |
| NH_4_HCO_3_ | 285.64±8.62a | 220.87±8.15a | 282.98±1.56a | 275.41±8.86a |
| Mean | 228.96* | 162.25* | 224.09* | 240.76* |
|  |  | **Matured leaves** |  |  |
| KNO_3_ | 144.98±0.36c | 107.04±0.04c | 129.67±0.39c | 127.65±0.09c |
| H_2_NCONH_2_ | 156.77±0.27b | 122.26±0.25ba | 160.84±0.20b | 131.95±0.08b |
| NaNO_3_ | 135.80±0.25dc | 105.65±0.13dc | 107.12±0.94d | 103.08±0.11d |
| NH_4_HCO_3_ | 166.36±0.13a | 130.84±0.19a | 189.12±0.35a | 141.22±0.27a |
| Mean | 150.98 | 116.45* | 146.69* | 125.98* |
|  |  | **Grains** |  |  |
| KNO_3_ | 66.40±0.96cb | 96.15±0.33c | 66.78±0.13c | 98.44±0.18c |
| H_2_NCONH_2_ | 69.69±0.68b | 105.91±0.17ab | 70.71±0.36ab | 108.41±0.20ab |
| NaNO_3_ | 47.53±0.15d | 84.51±0.59d | 58.44±0.19dc | 86.50±0.23d |
| NH_4_HCO_3_ | 87.87±0.14a | 111.91±0.14a | 74.24±0.30a | 117.35±0.50a |
| Mean 67.89** 99.62** 67.55** 102.68** | | | | |

Values sharing a common letter within the same variety do not differ significantly at P < 0.05 level. Means of the four rice cultivars followed by asterisk(s) (*, **) differ significantly at P < 0.05 and P < 0.01 level within the same variety respectively.

B385: Basmati 385, YJY: Yunjingyou, XYXZ: Xiangyaxiangzhan, DHX: Daohuaxiang
